# Enhanced epithelial to mesenchymal transition (EMT) and upregulated MYC in ectopic lesions contribute independently to endometriosis

**DOI:** 10.1186/s12958-015-0063-7

**Published:** 2015-07-22

**Authors:** Katharina Proestling, Peter Birner, Susanne Gamperl, Nadine Nirtl, Erika Marton, Gülen Yerlikaya, Rene Wenzl, Berthold Streubel, Heinrich Husslein

**Affiliations:** Department of Obstetrics and Gynecology, Medical University of Vienna, Waehringer Guertel 18-20, Vienna, 1090 Austria; Department of Pathology, Medical University of Vienna, Waehringer Guertel 18-20, Vienna, 1090 Austria; Department of Obstetrics and Gynecology, St. Michael’s Hospital, University of Toronto, 30 Bond street, Toronto, Ontorio M5B 1 W8 Canada

**Keywords:** Endometriosis, MYC, TWIST1, SNAIL, SLUG, EMT

## Abstract

**Background:**

Epithelial to mesenchymal transition (EMT) is a process in which epithelial cells lose polarity and cell-to-cell contacts and acquire the migratory and invasive abilities of mesenchymal cells. These abilities are thought to be prerequisites for the establishment of endometriotic lesions. A hallmark of EMT is the functional loss of E-cadherin (CDH1) expression in epithelial cells. TWIST1, a transcription factor that represses E-cadherin transcription, is among the EMT inducers. SNAIL, a zinc-finger transcription factor, and its close relative SLUG have similar properties to TWIST1 and are thus also EMT inducers. MYC, which is upregulated by estrogens in the uterus by an estrogen response cis-acting element (ERE) in its promoter, is associated with proliferation in endometriosis. The role of EMT and proliferation in the pathogenesis of endometriosis was evaluated by analyzing TWIST1, CDH1 and MYC expression.

**Methods:**

CDH1, TWIST1, SNAIL and SLUG mRNA expression was analyzed by qRT-PCR from 47 controls and 74 patients with endometriosis. Approximately 42 ectopic and 62 eutopic endometrial tissues, of which 30 were matched samples, were collected during the same surgical procedure. We evaluated TWIST1 and MYC protein expression by immunohistochemistry (IHC) in the epithelial and stromal tissue of 69 eutopic and 90 ectopic endometrium samples, of which 49 matched samples were analyzed from the same patient. Concordant expression of TWIST1/SNAIL/SLUG and CDH1 but also of TWIST1 and MYC was analyzed.

**Results:**

We found that TWIST1, SNAIL and SLUG are overexpressed (p < 0.001, p = 0.016 and p < 0.001) in endometriosis, while CDH1 expression was concordantly reduced in these samples (p < 0.001). Similar to TWIST1, the epithelial expression of MYC was also significantly enhanced in ectopic endometrium compared to eutopic tissues (p = 0.008). We found exclusive expression of either TWIST1 or MYC in the same samples (p = 0.003).

**Conclusions:**

Epithelial TWIST1 is overexpressed in endometriosis and may contribute to the formation of endometriotic lesions by inducing epithelial to mesenchymal transition, as CDH1 was reduced in ectopic lesions. We found exclusive expression of either TWIST1 or MYC in the same samples, indicating that EMT and proliferation contribute independently of each other to the formation of endometriotic lesions.

**Electronic supplementary material:**

The online version of this article (doi:10.1186/s12958-015-0063-7) contains supplementary material, which is available to authorized users.

## Background

Endometriosis is a benign gynecological disease characterized by the presence of functional endometrial glands and stroma outside the uterine cavity [1]. The precise etiology of endometriosis is unclear. One of the more widely accepted hypotheses is that endometriosis originates from the retrograde menstruation of endometrial cells that implant on peritoneal surfaces [2]. Although retrograde menstruation can be observed in many women, only a minority develop endometriosis. The success of the ectopic implantation seems to be dependent on several factors, including changes enabling endometrial cell migration, adhesion and invasive growth, as well as changes in anti-apoptotic signaling, angiogenesis and inflammatory response [3, 1, 4]. Epithelial to mesenchymal transition (EMT) is a process whereby epithelial cells lose polarity and cell-to-cell contacts and acquire the migratory and invasive abilities of mesenchymal cells [5]. These abilities might be prerequisites for the establishment of endometriotic lesions.

A hallmark of EMT is the functional loss of E-cadherin expression in epithelial cells. For endometriosis, studies on E-cadherin expression have led to contradictory results. While some studies reported a reduction of E-cadherin expression in endometriosis compared with the endometrium [6–9], others found no difference in E-cadherin expression in endometriosis compared with the endometrium [10–14]. Previous studies demonstrated that E-cadherin-negative epithelial cells were increased in peritoneal endometriosis compared with eutopic endometrium and that in vitro, E-cadherin-negative, N-cadherin-positive endometriotic epithelial cells showed invasive growth [15, 16]. Loss of E-cadherin expression together with a cadherin switch, in which E-cadherin is replaced by the expression of mesenchymal cadherins such as N-cadherin, is an important feature of EMT [5].

TWIST1, a highly conserved basic helix-loop-helix (bHLH) transcription factor that represses E-cadherin (CDH1) transcription, represents an EMT inducer and has been convincingly associated with tumor progression and the metastatic process [17–19].

SNAIL, a zinc-finger transcription factor, and its close relative SLUG have similar properties to TWIST1 and thus belong to the EMT inducers [14].

Factors that facilitate the survival and proliferation of misplaced endometrial cells may also contribute to the development of endometriosis. In endometriosis, epithelial and stromal cells show a lower number of apoptotic cells than in patients without endometriosis [20–22]. It has been hypothesized that the expression of the anti-apoptotic factor BCL-2 and the reduction of the pro-apoptotic factor BAX in endometriosis lesions allows for the survival of the tissue in ectopic sites [23, 24, 21, 22]. Increased expression of genes such as MYC, Cyclin D1, and Ki67 was shown to be upregulated in ectopic tissues, suggesting that lesions exhibit a higher proliferation rate [25, 26, 23, 27, 28]. Several studies investigating MYC expression in endometriosis observed increased MYC mRNA and protein expression in ectopic and eutopic endometrium from endometriosis patients [26, 29–31, 25].

Using qRT-PCR and immunohistochemistry (IHC), we analyzed the expression, localization and correlation of CDH1 and TWIST1, CDH1 and SNAIL, CDH1 and SLUG, and TWIST1 and MYC in more than 100 ectopic and eutopic endometrial tissues from the proliferative and secretory endometrium of women with endometriosis and in matched tissue samples from the same patients. At present, the concurrent expression of TWIST1 and MYC in the same sample and in paired analysis of eutopic and ectopic tissues of the same patient has never been evaluated.

## Methods

### Patients and tissue samples

Samples were collected between 2010 and 2014 and were analyzed under protocols approved by the institutional review board of the Medical University of Vienna (6th July 2010, reference number 545/2010). Signed informed consent was obtained from each participant of this study.

For qRT-PCR, tissue samples were obtained from 121 premenopausal women (mean age 32.3 ± 5.9 years) who underwent laparoscopic surgery at the certified Endometriosis Centre at the university-affiliated General Hospital of Vienna between 2010 and 2014 due to the suspicion of endometriosis with or without infertility. The 121 cases consisted of 74 patients with endometriosis and 47 control patients who also underwent hysteroscopy, including dilation and curettage, due to unexplained infertility. Among the 74 cases with endometriosis, we obtained matched samples of ectopic and eutopic endometrium in 30 cases, exclusively eutopic endometrium in 12 cases, and exclusively ectopic endometrium in 32 cases. The matched sample tissues were collected during the same surgical procedure. Endometriosis was diagnosed histologically in 62 patients and by visual inspection in 12 patients. Staging was performed according to the revised American Fertility Society (rAFS) classification guidelines (I, n = 10; II, n = 9; III, n = 25; IV, n = 30) [32]. Patients with malignant diseases of the ovaries or the endometrium were excluded. Ectopic lesions consisted of ovarian lesions (n = 40), peritoneal lesions (n = 13), and deep infiltrating lesions (n = 9). Characteristics of the study populations are provided in Additional file 1: Table S1.

For IHC, tissue samples were collected under the same conditions as for the qRT-PCR samples. The 160 cases consisted of 110 patients with endometriosis and 50 control patients who underwent dilation and curettage for benign indications. Among the 110 cases with endometriosis, we obtained matched samples of ectopic and eutopic endometrium in 49 cases, exclusively eutopic endometrium in 20 cases, and exclusively ectopic endometrium in 41 cases. Staging was performed according to the revised American Fertility Society (rAFS) classification guidelines (I, n = 17; II, n = 23; III, n = 22; IV, n = 25) [32]. Characteristics of the study populations are provided in Additional file 2: Table S2.

### Quantitative Real-Time PCR (qRT-PCR)

Briefly, total RNA was isolated from fresh frozen tissues with the Absolutely RNA miRNA Kit (Agilent) and reverse-transcribed with the SuperScript First-Strand Kit (Invitrogen) according to the manufacturers’ instructions. Each sample was analyzed by real-time PCR on an Applied Biosystems 7500 fast instrument, using gene-specific primers and fluorescent probes obtained from Applied Biosystems: CDH1, Hs_01023894_m1; TWIST1, Hs_01675818_m1; SNAIL, Hs_00195591_m1; SLUG, Hs_00950344_m1; GAPDH, Hs_99999905_m1 (control), and ACTB (control), Hs_99999903_m1. The mRNA levels of CDH1, TWIST1, SNAIL and SLUG were normalized to those of ACTB and GAPDH in each sample by subtracting the mean Ct (threshold cycle) values of the controls from the Ct value of CDH1, TWIST1, SNAIL and SLUG as described previously [33]. For binary analysis, the cutoff was set at 0.162 for CDH1 expression, at 0.031 for TWIST1 expression, at 0.0075 for SNAIL expression and at 0.156 for SLUG expression.

### Immunohistochemistry (IHC)

#### TWIST1

Immunohistochemical staining was performed on formalin-fixed, paraffin-embedded tissues. Three-micrometer-thick sections were cut and placed on glass slides. Heat antigen retrieval was performed in 10 mM Sodium Citrate Buffer pH6. Nonspecific background staining was blocked by incubation in H_2_O_2_ and with Ultra V Block (Thermo Scientific, Ultra Vision LP Kit, TL-060-HL) according to the protocol. The rabbit polyclonal IgG to humanTWIST amino acids 12–27 (Abcam, ab50581) was applied at a dilution of 1:1200 with Antibody Diluent with Background Reducing Components (Dako, S3022) and incubated overnight at 4 °C. The Ultra Vision LP Kit was used according to the protocol (Thermo Scientific, Ultra Vision LP Kit, TL-060-HL). Finally, all slides were incubated with DAB-Substrate (Dako, K346811) and counterstained in hematoxylin before they were dehydrated and mounted.

#### MYC

MYC IHC was performed with a professional staining system (AutostainerLink48, DAKO, Glostrup, Denmark) at the Department of Pathology in the Wilhelminen Hospital. Briefly, antigen retrieval was performed by boiling the slides in EnVision FLEX Target Retrieval Solution at high pH (Dako Kit, K8000) for 15 min at 97 °C. The blocking procedure was performed according to the protocol (Dako, K8000). The rabbit monoclonal IgG to human c-MYC [Y69] (Biocare, CME415AK, CK) was applied at a dilution of 1:100 with Renoir Red Diluent (Biocare) and incubated for 20 min at room temperature. The slides were incubated with polymer according to the protocol (Dako, K8000). Finally, all slides were incubated with DAB-Substrate (Dako, K8000) and counterstained in hematoxylin (Dako real hematoxylin, S2020) before they were dehydrated and mounted.

### Scoring and immunohistochemical analysis

**Fig. 1:**
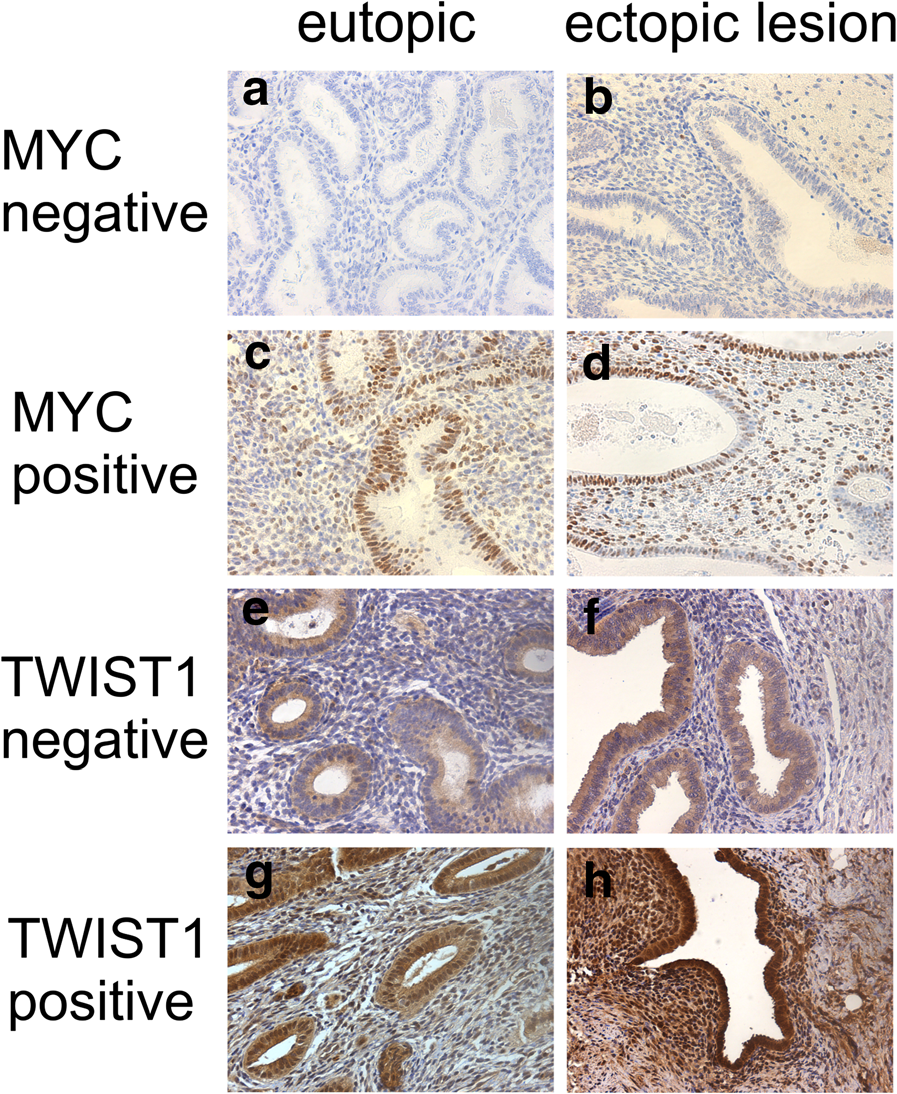
Immunohistochemical staining of MYC and TWIST1 in eutopic and ectopic endometrial tissue. Anti-MYC antibody was applied at a dilution of 1:100 and yielded negative (**a, b**) or positive (**c, d**) nuclear staining in eutopic (**a, c**) and ectopic (**b, d**) tissue. Anti-TWIST1 antibody was applied at a dilution of 1:1200 and yielded cytoplasmatic and nuclear staining. For evaluation, only nuclear staining was analyzed. Anti-TWIST1 antibody yielded negative (**e, f**) or positive (**g, h**) nuclear staining in eutopic (**e, g**) and ectopic (**f, h**) tissue. Magnification = 200x

Prior to immunohistochemistry, endometriotic lesions consisting of well-defined glandular epithelial and stromal cells were identified in hematoxylin-eosin-stained sections by a pathologist. Serial sections were cut from the chosen samples. A semiquantitative subjective scoring system to evaluate the localization, quantity and intensity of immunoreactivity was employed using light microscopy (200 × magnification). In each sample, the staining of glandular epithelial cells and stromal cells was scored separately. The intensity of the staining was scored using a four-point scoring scale (0, negative staining; 1, weak staining; 2 moderate staining, 3, strong staining). The percentage of positively stained cells was again scored using a four-point scoring scale (0, negative staining; 1, 1-35 % positive cells; 2, 36-70 % positive cells; 3, >67 % positive cells). The two scores were combined by multiplication to derive a final IHC score (0–9). For epithelial or stromal TWIST1 and epithelial MYC expression, a final score of ≥4 was regarded as positive, and for stromal MYC expression, a final score of ≥3 was regarded as positive (Fig. 1). Evaluations were performed by two blinded investigators. The outcomes analyzed by two experienced investigators showed statistical significance for the same results. An automatic quantitative analysis system was not robust/adequate for the analysis of our probes and was therefore not used. Positive and negative (without primary antibody) controls were run concurrently. The MYC protein was expressed in the nucleus of the epithelial and the stromal cells of eutopic and ectopic endometrium. TWIST expression was observed in the cytoplasm and the nucleus of epithelial and stromal cells. However, as a transcription factor, activated TWIST exerts its main function in the nucleus. Thus, for both factors, only the nuclear staining of epithelial and stromal cells was evaluated.

### Statistical analysis

Data were analyzed using SPSS (17.0). For association analyses, chi-squared tests were used. Wilcoxon and Mann Whitney U tests were used to compare the two groups. For correlation analyses, Spearman tests were used. For paired statistics, the McNemar Test and Wilcoxon Signed Ranks Test were used. A linear regression model was computed to describe associations between MYC, TWIST1, and cycle phase. We considered the subgroup analyses as exploratory and hence did not adjust for multiple testing, as recommended by Bender and Lange [34]. Statistical significance was defined as p < 0.05.

## Results

### Decrease of *CDH1* and increase of *TWIST1, SNAIL and SLUG* occur concordantly in ectopic lesions

**Fig. 2:**
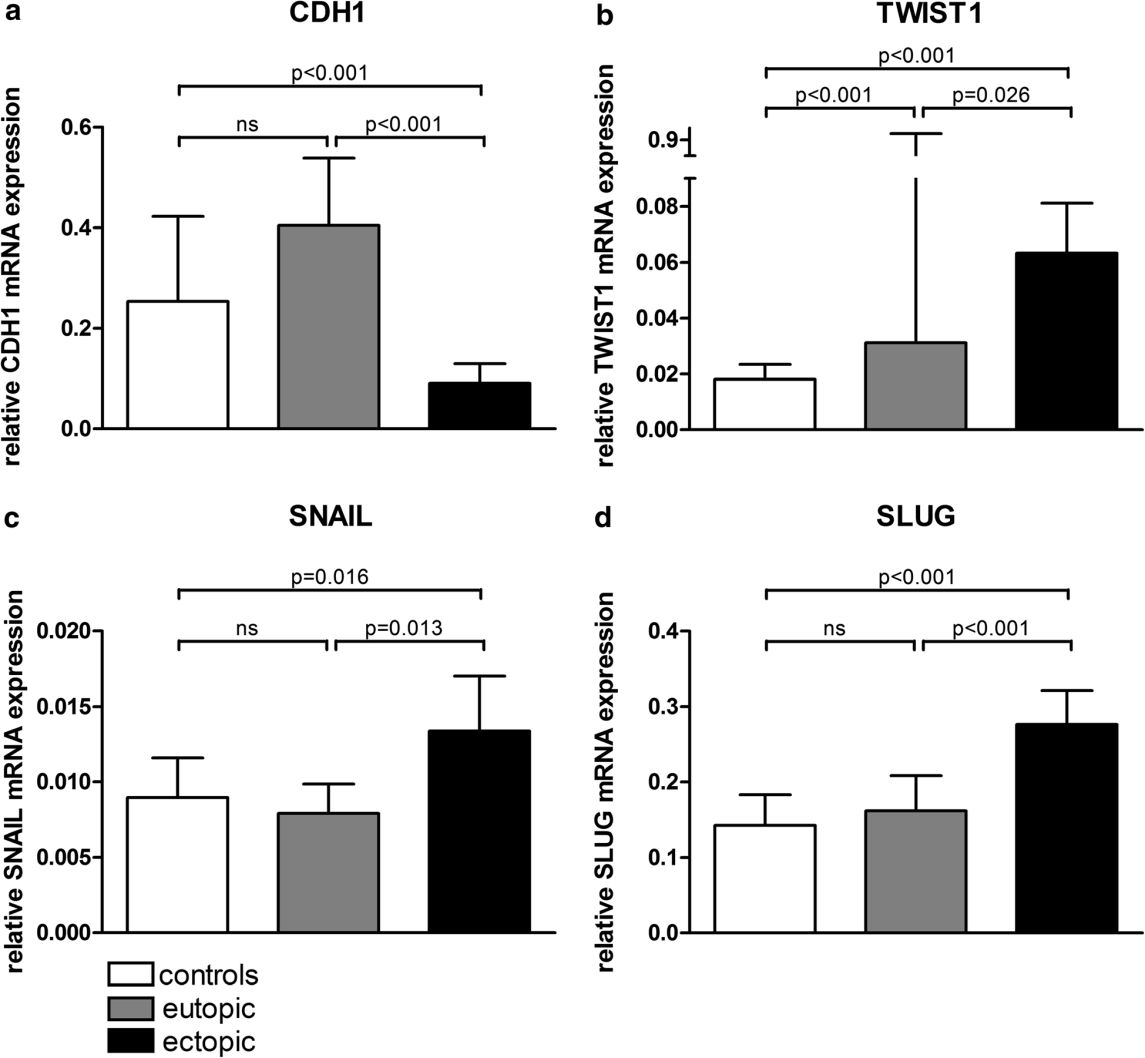
Bar graph of relative expression levels of *CDH1*, *TWIST1*, *SNAIL* and *SLUG*. Expression levels of *CDH1* mRNA (**a**) and *TWIST1* mRNA (**b**) are shown for controls (n = 45 and 47 for *CDH1* and *TWIST1*, respectively) and eutopic (n = 42) and ectopic endometrial samples (n = 62). Expression levels of *SNAIL* mRNA (**c**) and *SLUG* mRNA (**d**) are shown for controls (n = 47) and eutopic (n = 42) and ectopic endometrial samples (n = 62). Expression levels were normalized to ß-actin and GAPDH. All p-values were analyzed by Mann–Whitney U Tests

*CDH1* mRNA expression was significantly decreased in ectopic lesions compared to the eutopic gland epithelium of controls and patients in unpaired (both p < 0.001; Mann Whitney *U* Test, Fig. 2a) and paired samples (p < 0.001, McNemar Test, Table 1). In contrast, *TWIST1* expression was significantly increased in ectopic lesions compared to eutopic gland epithelium of controls and patients in unpaired (p < 0.001 and p = 0.026; Mann Whitney *U* Test, Fig. 2b) and paired samples (p = 0.049, McNemar Test, Table 1). *TWIST1* was also significantly more highly expressed in the eutopic endometrium of patients than in controls (p < 0.001; Mann Whitney *U* Test, Fig. 2b). In ectopic samples, most of the *CDH1*-negative samples (63.3 %) were concordantly positive for *TWIST1* expression (p < 0.001; McNemar Test, Table 2). In eutopic samples of controls and patients, many of the *CDH1*-positive samples were concordantly negative for *TWIST1* expression (64.3 % of controls and 48.5 % of patients, p < 0.001 and p = 0.012; McNemar Test, Table 2). In conclusion, *TWIST1* was upregulated, whereas *CDH1* was downregulated in ectopic tissues.

**Table 1:** *CDH1*, *TWIST1*, *SNAIL* and *SLUG* expressions in the eutopic and ectopic endometrium of the same patient

		**Ectopic** ***CDH1***					
		total	neg		pos		p-value
**Eutopic** ***CDH1***	neg	7	4	(57.1 %)	3	(42.9 %)	0.001
	pos	22	18	(81.8 %)	4	(18.2 %)	
		**Ectopic** ***TWIST1***					
		total	neg		pos		p-value
**Eutopic** ***TWIST1***	neg	15	2	(13.3 %)	13	(86.7 %)	0.049
	pos	14	4	(28.6 %)	10	(71.4 %)	
		**Ectopic** ***SNAIL***					
		total	neg		pos		p-value
**Eutopic** ***SNAIL***	neg	17	7	(41.2 %)	10	(58.8 %)	0.180
	pos	12	4	(33.3 %)	8	(66.7 %)	
		**Ectopic** ***SLUG***					
		total	neg		pos		p-value
**Eutopic** ***SLUG***	neg	20	7	(35 %)	13	(65 %)	0.007
	pos	9	2	(22.2 %)	7	(77.8 %)	

Numbers of patients in each of the indicated subgroups are shown. Numbers in parentheses indicate the fraction of patients (%) in each row in ectopic endometriotic lesions negative and positive for *CDH1*, *TWIST1, SNAIL or SLUG*. All p-values of subgroup comparisons were analyzed by the McNemar Test

**Table 2:** Correlation of *CDH1* and *TWIST1* expressions in control, eutopic and ectopic samples

**Controls**							
		*TWIST1*					
		total	neg		pos		p-value
*CDH1*	neg	17	17	(100 %)	0	(0.0 %)	<0.001
	pos	28	18	(64.3 %)	10	(35.7 %)	
**Eutopic**							
		*TWIST1*					
		total	neg		pos		p-value
*CDH1*	neg	9	5	(55.6 %)	4	(44.4 %)	0.012
	pos	33	16	(48.5 %)	17	(51.5 %)	
**Ectopic**							
		*TWIST1*					
		total	neg		pos		p-value
CDH1	neg	49	18	(36.7 %)	31	(63.3 %)	<0.001
	pos	13	0	(0.0 %)	13	(100 %)	

Numbers of patients in each of the indicated subgroups are shown. Numbers in parentheses indicate the fraction of patients (%) in each row negative and positive for *TWIST*. All p-values of subgroup comparisons were analyzed by the McNemar Test

*SNAIL* expression was significantly increased in ectopic lesions compared to the eutopic gland epithelium of controls and patients in unpaired (p = 0.016 and p = 0.013; Mann Whitney *U* Test, Fig. 2c) and paired samples (p = 0.180, McNemar Test, Table 1). In ectopic samples, most of the *CDH1*-negative samples (52.08 %) were concordantly positive for *TWIST1* expression (p < 0.001; McNemar Test, Table 3). In eutopic samples of patients, many of the *CDH1*-positive samples were concordantly negative for *SNAIL* expression (57.58 % of patients, p = 0.001; McNemar Test, Table 3). The expression of TWIST1 correlates positively with SNAIL expression in most samples (see Additional file 3: Table S4). In conclusion, *SNAIL* was upregulated whereas *CDH1* was downregulated in ectopic tissues.

**Table 3:** Correlation of *CDH1* and *SNAIL* expressions in control, eutopic and ectopic samples

**Controls**							
		*SNAIL*					
		total	neg		pos		p-value
*CDH1*	neg	19	14	(73.7 %)	5	(36.3 %)	0.210
	pos	28	11	(39.3 %)	17	(60.7 %)	
**Eutopic**							
		*SNAIL*					
		total	neg		pos		p-value
*CDH1*	neg	9	6	(66.7 %)	3	(33.3 %)	0.001
	pos	33	19	(57.6 %)	14	(42.4 %)	
**Ectopic**							
		*SNAIL*					
		total	neg		pos		p-value
CDH1	neg	48	23	(47.9 %)	25	(52.1 %)	<0.001
	pos	14	2	(14.3 %)	12	(85.7 %)	

Numbers of patients in each of the indicated subgroups are shown. Numbers in parentheses indicate the fraction of patients (%) in each row negative and positive for *SNAIL*. All p-values of subgroup comparisons were analyzed by the McNemar Test

Similar to *TWIST1* and *SNAIL* expression, *SLUG* expression was significantly increased in ectopic lesions compared to the eutopic gland epithelium of controls and patients in unpaired (both p < 0.001; Mann Whitney *U* Test, Fig. 2d) and paired samples (p = 0.007, McNemar Test, Table 1). In ectopic samples, most of the *CDH1*-negative samples (68.75 %) were concordantly positive for *SLUG* expression (p < 0.001; McNemar Test, Table 4). In eutopic samples of patients, many of the *CDH1*-positive samples were concordantly negative for *SLUG* expression (63.64 % of patients, p < 0.001; McNemar Test, Table 4). In ectopic tissue, the expression of TWIST1 correlates with SLUG expression (Additional file 4: Table S5). In conclusion, *SLUG* was upregulated whereas *CDH1* was downregulated in ectopic tissues.

**Table 4:** Correlation of *CDH1* and *SLUG* expressions in control, eutopic and ectopic samples

**Controls**							
		*SLUG*					
		total	neg		pos		p-value
*CDH1*	neg	19	15	(79.0 %)	4	(21.1 %)	0.019
	pos	28	15	(53.6 %)	13	(46.4 %)	
**Eutopic**							
		*SLUG*					
		total	neg		pos		p-value
*CDH1*	neg	9	7	(77.9 %)	2	(22.2 %)	<0.001
	pos	33	21	(63.6 %)	12	(36.4 %)	
**Ectopic**							
		*SLUG*					
		total	neg		pos		p-value
CDH1	neg	48	15	(31.3 %)	33	(68.8 %)	<0.001
	pos	14	2	(14.3 %)	12	(85.7 %)	

Numbers of patients in each of the indicated subgroups are shown. Numbers in parentheses indicate the fraction of patients (%) in each row negative and positive for *SLUG*. All p-values of subgroup comparisons were analyzed by the McNemar Test

### Increased TWIST1 and MYC in ectopic lesions

**Fig. 3:**
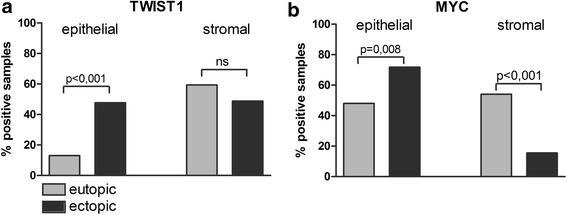
Expression and localization of TWIST1 and MYC in eutopic and ectopic endometrial tissue. IHC was used to analyze the protein expression of TWIST1 (**a**) and MYC (**b**). Results are expressed as the percentage of positively stained samples in eutopic (n = 69 and 50 for TWIST1 and MYC, respectively) and ectopic samples (n = 86 and 71 for TWIST1 and MYC, respectively). Epithelial and stromal expression was analyzed separately. All p-values of subgroup comparisons were analyzed by chi-squared tests

Epithelial TWIST1 expression was significantly more frequent in ectopic lesions compared to eutopic gland epithelium in unpaired (13.0 % vs. 47.7 %, p < 0.001; Chi^2^ Test, Fig. 3a) and paired samples (p < 0.001, McNemar Test, Table 5). Epithelial MYC expression was also more frequent in ectopic endometriotic lesions than in eutopic gland epithelium in unpaired samples (48.0 % vs. 71.8 %, p = 0.008, Chi^2^ Test, Fig. 3b). However, in paired analysis, no significant upregulation of MYC in ectopic samples was demonstrated (p = 0.180). In stromal cells, TWIST1 was not significantly differently expressed between eutopic and ectopic samples (Fig. 3a). In contrast, stromal MYC was expressed in only 15.5 % of the ectopic lesions, while more than 54 % of the eutopic endometrium samples showed positive stromal MYC staining (p < 0.001, Chi^2^ Test, Fig. 3b). Similarly, in paired analysis, stromal MYC expression was significantly enhanced in eutopic samples compared to the ectopic samples. Nearly 80 % of the samples showed stromal MYC positivity in eutopic samples and concordantly exhibited MYC negativity in ectopic samples of the same patient (p = 0.006, McNemar Test, Table 5). In conclusion, protein expression of MYC and TWIST1 was upregulated in glandular epithelium, whereas stromal MYC expression was downregulated in ectopic tissues.

**Table 5:** Epithelial and stromal TWIST1 and MYC expressions in the eutopic and ectopic endometrium of the same patient

		**Ectopic TWIST1**					
		total	neg		pos		p-value
**Eutopic Epithelial TWIST1**	neg	42	15	(35.7 %)	27	(64.3 %)	<0.001
	pos	5	3	(60.0 %)	2	(40.0 %)	
**Eutopic Stromal TWIST1**	neg	19	10	(52.6 %)	9	(47.4 %)	0.664
	pos	28	12	(42.9 %)	16	(57.1 %)	
		**Ectopic MYC**					
		total	neg		pos		p-value
**Eutopic Epithelial MYC**	neg	14	7	(50,0 %)	7	(50,0 %)	0.180
	pos	15	2	(13.3 %)	13	(86.7 %)	
**Eutopic Stromal MYC**	neg	15	14	(93.3 %)	1	(6.7 %)	0.006
	pos	14	11	(78.6 %)	3	(21.4 %)	

Numbers of patients in each of the indicated subgroups are shown. Numbers in parentheses indicate the fraction of patients (%) in each row in ectopic endometriotic lesions negative and positive for TWIST or MYC. All p-values of subgroup comparisons were analyzed by the McNemar Test

### TWIST1 and MYC are not concurrently expressed

Next we analyzed the concurrent expression of TWIST1 and MYC in eutopic and ectopic tissues. Most eutopic samples showing positive epithelial expression of MYC showed negative epithelial staining for TWIST1 (83.3 %, p = 0.002; McNemar test, Table 6). Similarly, in ectopic lesions, 62.5 % of samples with positive epithelial MYC staining showed MYC-negative epithelial cells (p = 0.003, McNemar test, Table 6). In stromal cells of ectopic tissues, 50.9 % of the samples with positive TWIST1 expression showed negative MYC expression (p < 0.001, McNemar test, Table 6). In conclusion, TWIST and MYC were not expressed concurrently in either eutopic endometrium or in ectopic lesions. It appears that the expression of one gene excludes the expression of the other.

**Table 6:** Correlation of epithelial and stromal TWIST1 and MYC expressions in eutopic and ectopic samples

**Eutopic endometrium**							
		TWIST1					
		total	neg		pos		p-value
Epithelial MYC	neg	26	22	(84.6 %)	4	(15.4 %)	0.002
	pos	24	20	(83.3 %)	4	(16.7 %)	
Stromal MYC	neg	23	9	(39.1 %)	14	(60.9 %)	0.115
	pos	27	6	(22.2 %)	21	(77.8 %)	
**Ectopic lesions**							
		TWIST1					
		total	neg		pos		p-value
Epithelial MYC	neg	20	10	(50.0 %)	10	(50.0 %)	0.003
	pos	48	30	(62.5 %)	18	(37.5 %)	
Stromal MYC	neg	57	28	(49.1 %)	29	(50.9 %)	<0.001
	pos	11	5	(45.5 %)	6	(54.5 %)	

Numbers of patients in each of the indicated subgroups are shown. Numbers in parentheses indicate the fraction of patients (%) in each row negative and positive for TWIST1. All p-values of subgroup comparisons were analyzed by the McNemar Test

### Epithelial MYC expression correlates with cycle phase

It is known that nuclear MYC expression is upregulated during the proliferative phase of the menstrual cycle [35, 26, 36]. Accordingly, in the eutopic endometrium of endometriosis patients, epithelial MYC expression is observed more frequently in patients in the proliferative cycle phase than in the secretory phase (p < 0.001, Chi^2^ test, see Additional file 5: Table S4). In endometriotic lesions, epithelial MYC expression is associated with the proliferative cycle phase, while negative MYC is significantly associated with the secretory phase of the patients (p = 0.046; see Additional file 5: Table S3). Using a linear regression model with epithelial MYC expression as the dependent variable and cycle phase and epithelial TWIST expression as independent variables, only cycle phase remained as an independent factor influencing epithelial MYC expression (p = 1.11×10^−6^, Coefficient −0.413, data not shown). No significant correlation was observed between the stromal MYC expression and the cycle phase of the patients in either eutopic or ectopic endometrium (see Additional file 5: Table S4). TWIST1 and *CDH1* expression did not correlate with the cycle phase in eutopic and in ectopic tissue (see Additional file 5: Table S3). In conclusion, a positive correlation between expression and menstrual cycle phase was found for epithelial MYC only.

*CDH1*, TWIST1 and MYC expression did not correlate with the rAFS staging classification (data not shown). When gene expression was analyzed according to the type of lesion (ie, ovarian, peritoneal, DIE), a significant difference was only found for the median *TWIST* expression between ovarian and DIE lesions (median 0.087 vs 0.059, p = 0.003).

## Discussion

In the present study, we were able to demonstrate that epithelial TWIST1, SNAIL and SLUG expression was overexpressed in ectopic lesions compared to eutopic endometrium glands. Correspondingly, in paired analysis of samples from the same patient, we found that in a significant proportion of samples, TWIST1, SNAIL and SLUG expression was negative in eutopic endometrium, whereas it was positive in ectopic lesions. We further showed overexpression of MYC in the glandular epithelium of endometriotic lesions compared to eutopic endometrium. Analysis of matched tissue samples revealed a trend towards more frequent epithelial MYC expression in ectopic lesions, although this did not reach significance. Few data exist that indicate a potential role of TWIST1 in the pathogenesis of endometriosis [14, 37]. Moreover, we showed a significant inverse expression between TWIST1 and CDH1 in controls and eutopic and ectopic tissue of patients. In addition, a significant inverse expression between SNAIL/ SLUG and CDH1 was observed in eutopic and ectopic tissue of patients. This finding suggests that TWIST1, SNAIL and SLUG might be important regulators of EMT in endometrium that is obviously upregulated in ectopic lesions. A study including patients with ovarian endometriosis analyzed mRNA expression of the stemness-related gene OCT4 and TWIST1 [37]. They reported an increased expression of OCT4 in ectopic endometrium and a positive correlation of OCT4 with TWIST1 [37]. The study that measured the mRNA expression of homogenized cells lacked discrimination between stromal and epithelial tissue. In our study of 110 endometriosis patients, we discriminated between epithelial and stromal protein expression by using IHC analysis for TWIST1 and MYC. We found that the epithelial expression of TWIST1 is significantly increased in ectopic endometrium while the stromal expression is reduced. Our findings suggest that the enhanced expression of TWIST1, SNAIL and SLUG in ectopic lesions plays a crucial role in the formation and maintenance of ectopic lesions in endometriosis. It can be hypothesized that the glandular epithelial cells lose polarity and cell-to-cell contacts by EMT and acquire migratory and invasive abilities to establish ectopic lesions.

In endometriosis, EMT is induced by multiple signals. For example, 17ß-estradiol (E2), which is known to be high in endometriotic tissue, has been shown to induce EMT in human endometrial epithelial cells through upregulation of the hepatocyte growth factor [38–40]. EMT can be induced by proinflammatory cytokines. One inflammatory mediator relevant in EMT is TGF-ß which is increased in peritoneal fluid of women with endometriosis [41]. TNF-α and IL-6 may synergistically nudge the TGF-ß signaling pathway towards EMT progression [42]. A significantly increased secretion of TNF-α and IL-6 in the culture media of peritoneal macrophages of endometriosis patients was found in response to E2 compared to nontreated macrophages [39]. Moreover, levels of IL-6 are higher in human endometrial stromal cells derived from the endometrial biopsies of women with endometriosis when compared with women without the disease [43]. TNF-α and IL-6 but also oxidative stress can promote NF-κB activation, which regulates the expression of Snail1, Slug, Twist, ZEB1, and ZEB2 [44, 42]. A recent study showed that iron overload leads to NF-κB activation in human endometrial stromal cells [44].

MYC is a well-known oncogene, and its function in tumor formation has been intensively studied. In endometriosis, the overexpression of MYC is also well established. Nevertheless, the role in pathogenesis is still unclear. MYC is upregulated in the ectopic and eutopic endometrium of patients with endometriosis when analyzed by reverse transcription PCR and IHC [26, 29–31, 25]. In the present study, we observed higher MYC expression in the glandular epithelium of endometriotic lesions compared to eutopic tissues, in concordance with Pellegrini et al. MYC overexpression suggests a higher proliferation rate in lesions than in eutopic tissues. In contrast to the epithelial expression of MYC, the stromal expression of MYC was predominantly negative in the ectopic endometrium.

Our observation of overexpressed MYC and TWIST1 in the epithelial cells of ectopic lesions prompted us to investigate a putative correlation of these two markers. In the present study, we were not able to detect the concurrent regulation of epithelial MYC and TWIST1 in the same sample in either eutopic or ectopic tissues. Actually, the exact opposite was true. We found a significant inverse expression of MYC and TWIST1 in paired samples. Thus, we excluded a simultaneous upregulation of TWIST1 and MYC that may orchestrate the cellular changes associated with invasion and proliferation in endometriosis. It seems that a high expression of TWIST1, which was shown to be associated with the stemness marker OCT4 in endometriosis, excludes a high expression of MYC, which is associated with proliferation [37] in endometriosis. TWIST1 has also been shown to be an important regulator of stemness in epithelial ovarian cancer [45, 46]. Thus, in ectopic lesions with high epithelial MYC expression, the additional upregulation of TWIST1 appears to provide no further advantage for the cell and vice versa. There are several other theories concerning the pathogenesis of endometriosis. Some studies revealed the presence of adult stem cells in the basalis but also functionalis layers of the human endometrium [47–50]. These endometrium-derived stem cells, which are distributed by retrograde menstrual efflux, may also contribute to the establishment of ectopic endometriotic lesions [50–54]. The monoclonal origin of some endometriotic lesions, long-time culture properties of cell clones established from endometriotic lesions, and the isolation of progenitor cells from menstrual blood support this hypothesis [55–60]. Another theory concerning the pathogenesis of endometriosis is the coelomic metaplasia theory, which proposes that mesothelial cells on the ovary or pelvis change to endometriotic gland cells [61, 62].

The observation of increased MYC expression during the proliferative cycle phase has already been reported [35, 26, 36]. In utero, MYC is upregulated by estrogens by an estrogen response cis-acting element (ERE) in its promotor [63–65]. Endometriotic lesions show increased production of estradiol and higher concentrations of estradiol have been detected in the peritoneal fluid of women with endometriosis than in that of normal controls [66]. Compared to estrogen receptor (ER) α, ERß is expressed in markedly higher levels in ectopic lesions than in eutopic tissue. [67]. Deficient methylation of the ERß gene promotor has been suggested to result in pathological overexpression of ERß in endometriosis, which in turn represses ERα expression [67]. Previous studies showed a remarkably elevation of ERß mRNA and protein expression relative to the normal endometrium [68, 25]. In addition, the eutopic endometrium of women with endometriosis have elevated ERß expression when compared with the endometrium of healthy women [69–71], suggesting that high levels of ERß in the endometrium may predispose women to endometriosis. Increased MYC expression might be due to enhanced local estrogen levels in endometriotic lesions [72, 63, 65]. In this study, we showed a significant correlation between MYC expression and the proliferative menstrual cycle phase of the women with high estrogen levels. In contrast, we confirmed previous findings that the expression of TWIST1 was not associated with cycle phase [14].

## Conclusions

We showed a significant inverse expression between TWIST1 and CDH1 in controls and eutopic and ectopic tissue of endometriosis patients. Furthermore, a significant inverse expression between SNAIL/ SLUG and CDH1 was observed in eutopic and ectopic tissue of patients. These findings suggest that TWIST1, SNAIL and SLUG might be important regulators of EMT in endometrium. Moreover, EMT seems to be enhanced in ectopic lesions compared to eutopic tissue. The results reported herein show increased expression of epithelial TWIST1 and MYC in ectopic endometrium compared to eutopic endometrium in paired and unpaired analysis. Although both were upregulated, TWIST1 and MYC were not expressed concurrently, which suggest that in cells with high MYC expression, an additional upregulation of TWIST1 and vice versa seems to provide no further advantage for the development of endometriosis. The transcription factor TWIST1 and the oncogene MYC seem to contribute independently of each other to the formation of endometriotic lesions.

## Additional files

Additional file 1: Table S1. Description of the EMMA study population. Additional file 2: Table S2. Description of the IHC study population. Additional file 3: Table S4. Correlation of *TWIST* and *SNAIL* expressions in control, eutopic and ectopic samples. Additional file 4: Table S5. Correlation of *TWIST* and *SLUG* expressions in control, eutopic and ectopic samples. Additional file 5: Table S3. CDH1, TWIST and MYC expression in the proliferative and secretory phases of eutopic and ectopic tissue.
