# Supplementary material for: Enhanced epithelial to mesenchymal transition (EMT) and upregulated MYC in ectopic lesions contribute independently to endometriosis
Source: Reprod Biol Endocrinol. 2015 Jul 22;13:75. doi: 10.1186/s12958-015-0063-7 (PMC4511248; doi:10.1186/s12958-015-0063-7)
Supplement: Additional file 1: Table S1. — Description of the EMMA study population. [file 12958_2015_63_MOESM1_ESM.docx]

**Additional file 1, Supplemental Table S1**Description of the EMMA study population

|  | | **total** | **Controls (n=47)** | | **Eutopic (n=42)** | | **Ectopic (n=62)** | |
| --- | --- | --- | --- | --- | --- | --- | --- | --- |
| Age (years) |  | 151 | 34.11± 6.2 | | 31.9 ± 6.2 | | 31.3 ± 5.3 | |
| Cycle Phase | Proliferative | 59 | 14 | (23.7%) | 15 | (25.4%) | 30 | (50.8%) |
|  | Secretory | 72 | 28 | (38.9%) | 21 | (29.2%) | 23 | (31.9%) |
|  | na | 20 | 5 | (25.0%) | 6 | (30.0%) | 9 | (45.0%) |
| Staging | I or II | 25 |  |  | 16 | (64.0%) | 9 | (36.0%) |
|  | III or IV | 79 |  |  | 26 | (32.9%) | 53 | (67.1%) |
|  | na | 47 | 47 | (100%) | 0 | (0%) | 0 | (0%) |

Numbers of patients in each of the indicated subgroups are shown. Numbers in parentheses indicate the fraction of patients (%) in each column in the proliferative and secretory cycle phases or with low and high stages [28]. na, status not available.
