# Supplementary material for: Enhanced epithelial to mesenchymal transition (EMT) and upregulated MYC in ectopic lesions contribute independently to endometriosis
Source: Reprod Biol Endocrinol. 2015 Jul 22;13:75. doi: 10.1186/s12958-015-0063-7 (PMC4511248; doi:10.1186/s12958-015-0063-7)
Supplement: Additional file 2: Table S2. — Description of the IHC study population. [file 12958_2015_63_MOESM2_ESM.docx]

**Additional file 2, Supplemental Table S2**Description of the IHC study population

|  | | **total** | **Controls (n=50)** | | **Eutopic (n=69)** | | **Ectopic (n=90)** | |
| --- | --- | --- | --- | --- | --- | --- | --- | --- |
| Age (years) |  | 209 | 34.9 ± 5.8 | | 33.1 ± 6.1 | | 34.7 ± 6.8 | |
| Cycle Phase | Proliferative | 99 | 24 | (24.2%) | 31 | (31.3%) | 44 | (44.4%) |
|  | Secretory | 92 | 20 | (21.7%) | 37 | (40.2%) | 35 | (38.0%) |
|  | na | 18 | 6 | (33.3%) | 1 | (5.6%) | 11 | (61.1%) |
| Staging | I or II | 62 |  |  | 22 | (35.5%) | 40 | (64.5%) |
|  | III or IV | 72 |  |  | 25 | (34.7%) | 47 | (65.3%) |
|  | na | 75 | 50 | (66.7%) | 22 | (29.3%) | 3 | (4.0%) |

Numbers of patients in each of the indicated subgroups are shown. Numbers in parentheses indicate the fraction of patients (%) in each column in the proliferative and secretory cycle phase or with low and high stages [28]. na, status not available.
