# Supplementary material for: Enhanced epithelial to mesenchymal transition (EMT) and upregulated MYC in ectopic lesions contribute independently to endometriosis
Source: Reprod Biol Endocrinol. 2015 Jul 22;13:75. doi: 10.1186/s12958-015-0063-7 (PMC4511248; doi:10.1186/s12958-015-0063-7)
Supplement: Additional file 3: Table S4. — Correlation of TWIST and SNAIL expressions in control, eutopic and ectopic samples. [file 12958_2015_63_MOESM3_ESM.docx]

**Additional file 3, Supplemental Table S4**Correlation of *TWIST* and *SNAIL* expressions in control, eutopic and ectopic samples

| **Controls** | | | | | | | |
| --- | --- | --- | --- | --- | --- | --- | --- |
|  | | *SNAIL* | | | | | |
|  |  | total | neg | | pos | | p-value |
| *TWIST1* | neg | 36 | 22 | (61.1%) | 14 | (38.9%) | 0.013 |
|  | pos | 11 | 3 | (27.3%) | 8 | (72.7%) |  |
| **Eutopic** | | | | | | | |
|  | | *SNAIL* | | | | | |
|  |  | total | neg | | pos | | p-value |
| *TWIST1* | neg | 21 | 15 | (71.4%) | 6 | (28.6%) | 0.454 |
|  | pos | 21 | 10 | (47.6%) | 11 | (52.4%) |  |
| **Ectopic** | | | | | | | |
|  | | *SNAIL* | | | | | |
|  |  | total | neg | | pos | | p-value |
| TWIST1 | neg | 18 | 11 | (61.1%) | 7 | (38.9%) | 0.189 |
|  | pos | 44 | 14 | (14.3%) | 30 | (68.2%) |  |

Numbers of patients in each of the indicated subgroups are shown. Numbers in parentheses indicate the fraction of patients (%) in each row negative and positive for *SNAIL*. All p-values of subgroup comparisons were analyzed by the McNemar Test.
