# Supplementary material for: Enhanced epithelial to mesenchymal transition (EMT) and upregulated MYC in ectopic lesions contribute independently to endometriosis
Source: Reprod Biol Endocrinol. 2015 Jul 22;13:75. doi: 10.1186/s12958-015-0063-7 (PMC4511248; doi:10.1186/s12958-015-0063-7)
Supplement: Additional file 4: Table S5. — Correlation of TWIST and SLUG expressions in control, eutopic and ectopic samples. [file 12958_2015_63_MOESM4_ESM.docx]

**Additional file 4, Supplemental Table S5**Correlation of *TWIST* and *SLUG* expressions in control, eutopic and ectopic samples

| **Controls** | | | | | | | |
| --- | --- | --- | --- | --- | --- | --- | --- |
|  | | *SLUG* | | | | | |
|  |  | total | neg | | pos | | p-value |
| *TWIST1* | neg | 36 | 23 | (63.9%) | 13 | (36.1%) | 0.263 |
|  | pos | 11 | 7 | (63.6%) | 4 | (36.4%) |  |
| **Eutopic** | | | | | | | |
|  | | *SLUG* | | | | | |
|  |  | total | neg | | pos | | p-value |
| *TWIST1* | neg | 21 | 12 | (57.1%) | 9 | (42.9%) | 0.230 |
|  | pos | 21 | 16 | (76.2%) | 5 | (23.8%) |  |
| **Ectopic** | | | | | | | |
|  | | *SLUG* | | | | | |
|  |  | total | neg | | pos | | p-value |
| TWIST1 | neg | 18 | 8 | (44.5%) | 10 | (55.6%) | 1.000 |
|  | pos | 44 | 9 | (20.5%) | 35 | (79.6%) |  |

Numbers of patients in each of the indicated subgroups are shown. Numbers in parentheses indicate the fraction of patients (%) in each row negative and positive for *SLUG*. All p-values of subgroup comparisons were analyzed by the McNemar Test.
