# Supplementary material for: Enhanced epithelial to mesenchymal transition (EMT) and upregulated MYC in ectopic lesions contribute independently to endometriosis
Source: Reprod Biol Endocrinol. 2015 Jul 22;13:75. doi: 10.1186/s12958-015-0063-7 (PMC4511248; doi:10.1186/s12958-015-0063-7)
Supplement: Additional file 5: Table S3. — CDH1, TWIST and MYC expression in the proliferative and secretory phases of eutopic and ectopic tissue. [file 12958_2015_63_MOESM5_ESM.docx]

**Additional file 5, Supplemental Table S3**CDH1, TWIST and MYC expression in the proliferative and secretory phases of eutopic and ectopic tissue.

| **IHC – Epithelial Expression** | | | | | | | |
| --- | --- | --- | --- | --- | --- | --- | --- |
|  | | MYC | | | | | |
|  |  | total | negative | | positive | | p-value |
| Eutopic endometrium | Proliferative Phase | 24 | 5 | (20.8%) | 19 | (79.2%) | <0.001 |
|  | Secretory Phase | 26 | 21 | (80.8%) | 5 | (19.2%) |  |
| Ectopic endometrium | Proliferative Phase | 37 | 8 | (21.6%) | 29 | (78.4%) | 0.046 |
|  | Secretory Phase | 24 | 11 | (45.8%) | 13 | (54.2%) |  |
|  | | TWIST | | | | | |
| Eutopic endometrium | Proliferative Phase | 31 | 27 | (87.1%) | 4 | (12.9%) | 0.941 |
|  | Secretory Phase | 37 | 32 | (86.5%) | 5 | (13.5%) |  |
| Ectopic endometrium | Proliferative Phase | 44 | 27 | (61.4%) | 17 | (38.6%) | 0.130 |
|  | Secretory Phase | 34 | 15 | (44.1%) | 19 | (55.9%) |  |
| **IHC – Stromal Expression** | | | | | | | |
|  | | MYC | | | | | |
| Eutopic endometrium | Proliferative Phase | 24 | 13 | (54.2%) | 11 | (45.8%) | 0.266 |
|  | Secretory Phase | 26 | 10 | (38.5%) | 16 | (61.5%) |  |
| Ectopic endometrium | Proliferative Phase | 37 | 34 | (91.9%) | 3 | (8.1%) | 0.136 |
|  | Secretory Phase | 24 | 18 | (75.0%) | 6 | (25.0%) |  |
|  | | TWIST | | | | | |
| Eutopic endometrium | Proliferative Phase | 31 | 11 | (35.5%) | 20 | (64.5%) | 0.515 |
|  | Secretory Phase | 37 | 16 | (43.2%) | 21 | (56.8%) |  |
| Ectopic endometrium | Proliferative Phase | 44 | 22 | (50.0%) | 22 | (50.0%) | 0.797 |
|  | Secretory Phase | 34 | 18 | (52.9%) | 16 | (47.1%) |  |
| **mRNA Expression** | | | | | | | |
|  |  | *CDH1* | | | | | |
| Eutopic endometrium | Proliferative Phase | 15 | 4 | (26.7%) | 11 | (73.3%) | 0.210 |
|  | Secretory Phase | 21 | 2 | (9.5%) | 19 | (90.5%) |  |
| Ectopic endometrium | Proliferative Phase | 30 | 25 | (83.3%) | 5 | (16.7%) | 0.730 |
|  | Secretory Phase | 23 | 18 | (78.3%) | 5 | (21.7%) |  |

Numbers of patients in each of the indicated subgroups are shown. Numbers in parentheses indicate the fraction of patients (%) in each column in the proliferative and secretory menstrual cycle phases. All p-values of subgroup comparisons were analyzed by chi-squared tests.
